# German version of the Cornell Musculoskeletal Discomfort Questionnaire (CMDQ): translation and validation

**DOI:** 10.1186/s12995-016-0100-2

**Published:** 2016-03-25

**Authors:** Steffi Kreuzfeld, Reingard Seibt, Mohit Kumar, Annika Rieger, Regina Stoll

**Affiliations:** Institute for Preventive Medicine of the Rostock University Medical Center, St.-Georg-Str. 108, D-18055 Rostock, Germany; Institute and Policlinics for Occupational and Social Medicine, Dresden University of Technology, Fetscherstr. 74, D-01307 Dresden, Germany; Center for Life Science Automation, University of Rostock, F.-Barnewitz-Str. 8, D-18055 Rostock, Germany

**Keywords:** Musculoskeletal disorders, Pain assessment, Questionnaires, Validation study, Work-related health promotion, Ergonomics, Prevention, Muskel-Skelett-Erkrankungen, Schmerzerfassung, Fragebögen, Validierung, Arbeitsplatzbezogene Gesundheitsförderung, Ergonomie, Prävention

## Abstract

**Background:**

Musculoskeletal disorders are a public health problem with significant effects on work ability. In the context of the promotion and prevention of work-related health, there is a need for valid, simple, time-saving and universally applicable methods for the assessment of musculoskeletal pain and complaints. The aim of this study was the translation of the English Cornell Musculoskeletal Discomfort Questionnaire (CMDQ) into German and the validation of the German version.

**Methods:**

The linguistic and cultural adaption of the CMDQ into German (D-CMDQ) followed international guidelines. The adapted pre-version was initially tested in terms of comprehensibility on 44 persons with different educational and occupational backgrounds. The questionnaire was validated further on 68 employees with the reference of an 11-point Numeric Rating Scale (Cohen’s Kappa and Spearman’s rank correlation coefficients). Finally, reliability (Cohen’s Kappa) and internal consistency (Cronbach’s alpha) were verified.

**Results:**

The D-CMDQ meets the requirements for comprehensibility and demonstrated good validity: The values of Cohen’s Kappa and Spearman’s rank correlation coefficient obtained substantial to excellent agreement, with one exception. The Kappa values for the test-retest reliability were mainly in the moderate to substantial range whilst taking the prevalence effect into account. The internal consistency was proven satisfactory.

**Conclusions:**

The D-CMDQ meets the psychometric requirements for questionnaires. A clear one-sided presentation of body areas enables the time-saving assessment of musculoskeletal complaints and their effects on work ability. As a result, a broad application in the German-speaking world for different occupational groups seems possible, whether performing physical, manually repetitive or sedentary work.

## Kurzfassung

**Hintergrund:**

Muskel-Skelett-Erkrankungen (MSD) stellen ein häufiges Gesundheitsproblem mit erheblichen Auswirkungen auf die Arbeitsfähigkeit dar. Im Kontext arbeitsplatzbezogener Gesundheitsförderung werden valide Verfahren benötigt, die sowohl das Ausmaß der Beschwerden als auch die Auswirkungen auf die Arbeitsfähigkeit und potenzielle Interventionseffekte zeitsparend erfassen. Diese Verfahren sollten möglichst universell, d.h. in Berufen mit unterschiedlichen körperlichen und mentalen Anforderungen einsetzbar sein. Das Ziel der Studie bestand darin, den als geeignet erscheinenden englischsprachigen Cornell Musculoskeletal Discomfort Questionnaire (CMDQ) ins Deutsche zu übersetzen und anschließend die psychometrische Güte der deutschen Version (D-CMDQ) zu überprüfen.

**Methoden:**

Der CMDQ wurde durch Vorwärts- und Rückwärtsübersetzung entsprechend internationaler Leitlinien linguistisch und kulturell an die deutsche Sprache angepasst. Anschließend wurden Klarheit und Verständlichkeit des adaptierten Fragebogens an 44 Personen unterschiedlichen Alters und Bildungsgrades getestet. Die Überprüfung der Testgüte der deutschen Version erfolgte an 68 Personen aus unterschiedlichen Berufen. Dabei wurde für die Validität das Ausmaß der Übereinstimmung mit einer 11-stufigen numerischen Schmerzskala anhand von Rangkorrelationskoeffizienten nach Spearman bzw. Cohen‘s Kappa ermittelt. Zusätzlich wurde die Test-Retest Reliabilität über die Kalkulation von Kappa-Koeffizienten und die interne Konsistenz mittels Cronbach’s Alpha getestet.

**Ergebnisse:**

Der D-CMDQ zeigte eine gute Validität: Cohen‘s Kappa and Spearman’s Korrelationskoeffizienten erreichten bis auf eine Ausnahme ein substantielles bis perfektes Niveau. Die Test-Retest Reliabilität lag nach Berücksichtigung des Prävalenzeffektes (relativer Anteil von Schmerzangaben in einer Körperregion) überwiegend im moderaten bis substanziellen Bereich. Die interne Konsistenz kann als gut eingeschätzt werden.

**Schlussfolgerungen:**

Die deutsche Version des CMDQ erfüllt die psychometrischen Anforderungen an Fragebögen. Übersichtlichkeit und Testökonomie erlauben die Anwendung in diversen Berufsgruppen, unabhängig davon, ob überwiegend körperlich schwer, manuell repetitiv oder sitzend gearbeitet wird. Damit steht für den deutschsprachigen Raum ein universelles Instrument zur Erfassung von Muskel-Skelett-Beschwerden in Arbeitsmedizin und Prävention zur Verfügung. Die Übersetzung in weitere Sprachen wäre in Bezug auf zukünftige, länderübergreifende Forschungsprojekte überaus wünschenswert.

## Background

Musculoskeletal disorders and complaints (MSD) are a major public health problem [1]. They often lead to an incapacity to carry out work, cause high medical costs, and constitute an economic burden on society [2, 3]. In Germany, in 2012, MSD caused almost one fourth of all sick leave (23.4 %) and thus led to a loss of approximately 21 billion Euros in gross value added [4]. For years, there has been observable absenteeism especially in occupational groups with high physical strain and rather modest remuneration. This absenteeism has frequently been caused by MSD and has lasted particularly long [5, 6]. Sectors which are particularly affected are the construction industry, as well as agriculture and forestry. However, musculoskeletal complaints play a major role for office workers as well [7]. Independent of occurring physical stress, psychosocial stress such as high job demands, low job control, and low social support may increase the risk of MSD [1, 8, 9].

MSD can have multiple causes and thus offer a wide range of preventive approaches consisting of ergonomic, work-organizational and psychosocial measures. Assessment tools are needed which are brief, valid and reliable to monitor the effectiveness of such preventive approaches, and should be applicable in a wide variety of settings in order to determine the impact of MSD on the current work ability.

A common and valid method for the acquisition of pain intensity is the use of one-dimensional pain scales (such as numeric rating scales, verbal rating scales, visual analogue scales) which can be applied to different anatomical regions [10, 11]. However, these scales do not consider functional aspects, such as occupational activities. Commonly used questionnaires with functional outcomes are for instance the Roland-Morris Disability Questionnaire or the Oswestry Disability Index [12, 13]. These tools, however, are limited to chronic low back pain and impairments in daily life. Other questionnaires dealing with office work focus solely on complaints in the arm and shoulder region as well as the neck area, as for example the RSI-QuickScan [14]. The Brief Pain Inventory [15] includes one pain interference item addressing work issues.

As opposed to the RSI-QuickScan, the commonly used Nordic Musculoskeletal Questionnaire (NMQ) acquires information on the presence of musculoskeletal complaints in nine relevant anatomical regions from the neck to the feet [16]. For the specific sections of the lower back, neck and shoulder region, the questionnaire additionally acquires information on the degree of pain as well as on the consequences of the disorder. In recent years several adaptations of the NMQ have been published including that of one-page versions [17].

**Fig. 1:**
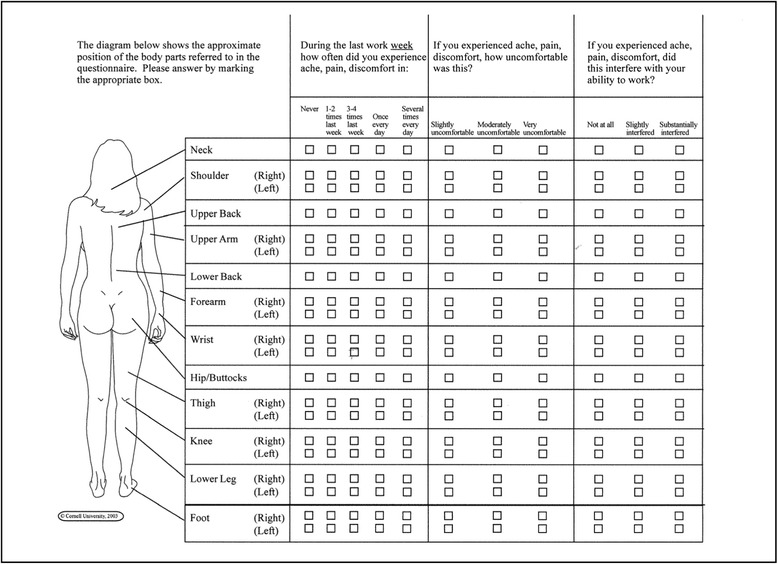
Female version of the originally CMDQ for standing worker

Compared with the above-mentioned questionnaires, the Cornell Musculoskeletal Discomfort Questionnaire (CMDQ) [18], combines the frequency and the intensity of musculoskeletal pain and complaints with work-related impairments for 20 body regions in a chart on only one page (see Fig. 1). The CMDQ thus meets the conditions of a good test economy: little time needed to perform, analyze and interpret the test, low material consumption, easy to use and the suitability for group testing. Furthermore, the CMDQ is applicable not only for workers with back pain, but also for any pain and complaints in other body regions. In this way it is universally usable for a wide range of professions as it allows the acquisition of respective information on the functional aspects of office work and physically demanding work.

The CMDQ has been used for the evaluation of intervention studies concerning office work [19, 20], in the health care system at work places for medical diagnostics [21], and in the field of nursing [22]. The questionnaire was originally created in English. There are already validated translations of the CMDQ in Turkish [23] and Farsi [24].

For its usage in German-speaking countries as well as for the application of transnational research projects, the objective of our study was the translation of the Cornell Musculoskeletal Discomfort Questionnaire into German and the adaptation and validation of the German version (D-CMDQ).

## Methods

### Cross-cultural adaptation

In order to establish the cultural equivalence of the original version of the CMDQ, we followed previously published guidelines for translation and cross-cultural adaptation of health status measures [25, 26]. The questionnaire was translated from English into German by two professional translators who worked independent of each other and whose native language is German. One of them was familiar with the concept of the questionnaire.

Then the written reports were discussed in a consensus panel between the two translators and a third person. All discrepancies in the written reports were checked against the original questionnaire and against each other regarding content analogousness, and critically judged in terms of everyday language to arrive at the preliminary version.

The translation of this version back into the original language was done by another professional translator whose native language is English and by a German physician who has been living in the USA for 25 years. Afterwards, any discrepancy between the original version, the translation, and the backwards translation were discussed on the basis of a structured interview in committee. Two translators, two occupational physicians, and two psychologists who were familiar with the intent of the measure and the concepts attended the committee. Again, first the semantic equivalence was checked and then all alternative suggestions were compared concerning unambiguity and popularity. Part of the review was also the instruction and the equivalence of steps in the scales. Subsequently, an interim final version was developed.

**Fig. 2:**
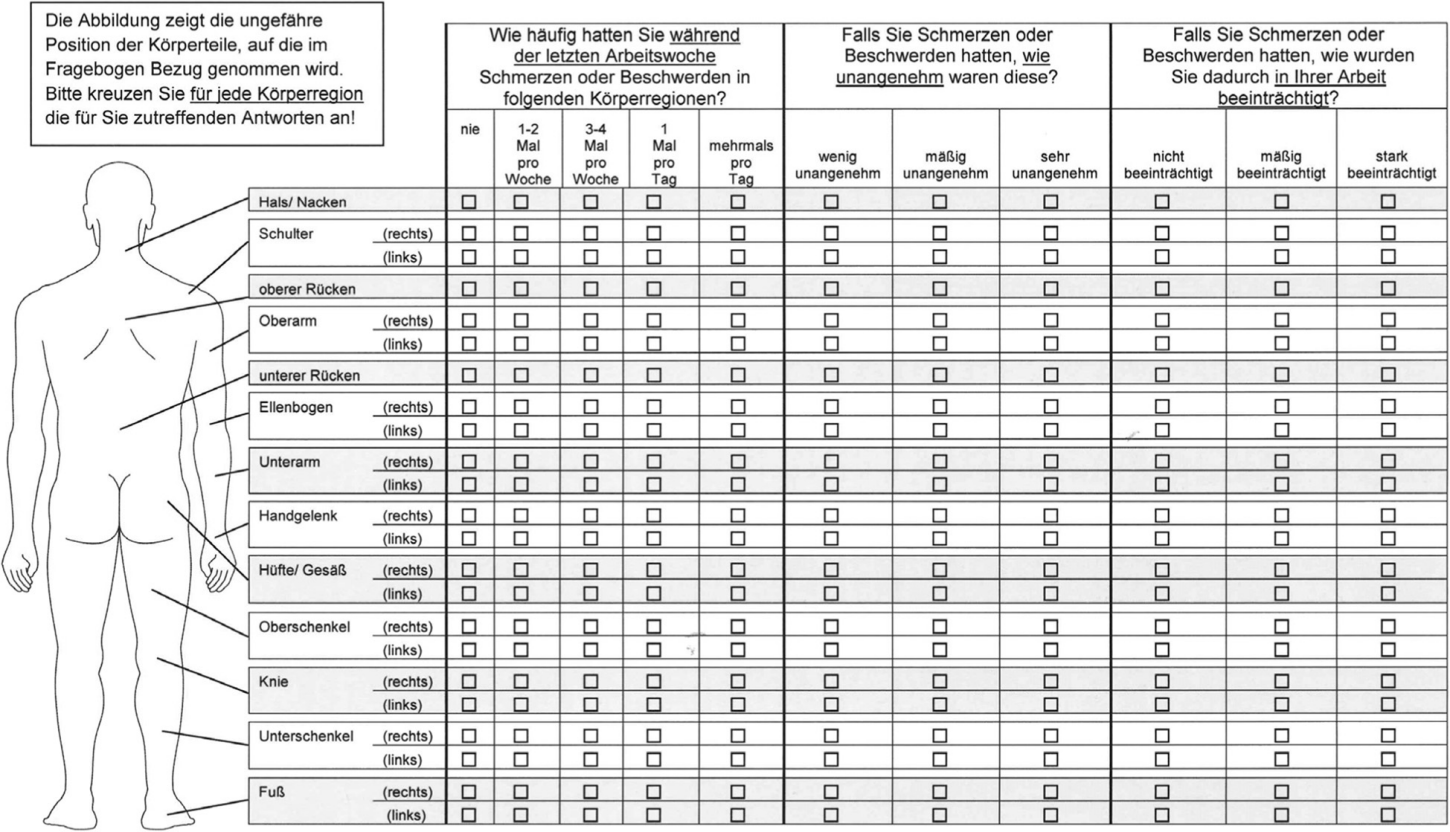
Male version of the modified D-CMDQ

**Fig. 3:**
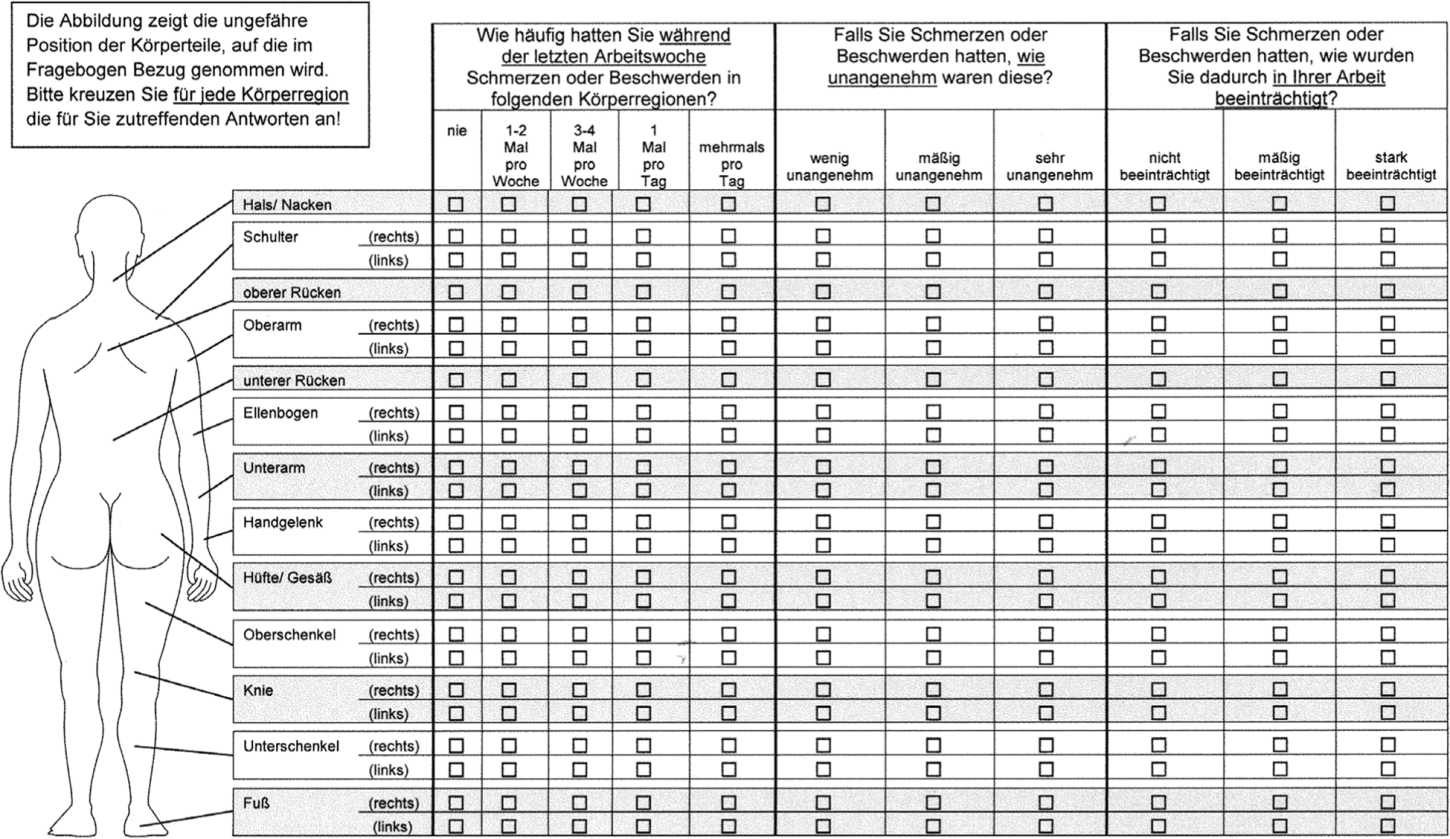
Female version of the modified D-CMDQ

Key words were then underlined within the scales in order to improve the questionnaire’s clarity. In a final step, the questionnaire’s graphics were revised (see Figs. 2 and 3). In an earlier validation study [23], errors occurred during questionnaire completion due to mistakes in the horizontal dimension within the questionnaire. The lines representing single anatomical regions were thus marked off by gray shades in the new version.

### Pretest

The German version of the CMDQ (D-CMDQ) was pretested on 44 subjects because a sample size between 30 and 50 participants enhances the probability of detecting even rare problems, e.g. unclear questions, unfamiliar words or ambiguous syntax [27]. The inclusion criteria covered 18- to 67- year-old native German speakers in either a regular job or in an academic or professional education. Non-native German speakers were excluded from the whole study. 59.1 % of the participants were females. The pretest’s main purpose was to validate the comprehensibility of the adapted version, thus the pretest included subjects with different educational backgrounds and different occupations, for example kitchen porters, cleaners, office employees, musicians, students, and research assistants. The proportions of the subjects with high, intermediate, and low educational status were about 47.7, 31.8, and 20.4 %, respectively. The participants were contacted in their respective professional environments, were informed both orally and in writing about the purpose of the research study and data protection, and were asked to indicate occurring difficulties with regard to the instructions, the questionnaire’s structure, or the comprehensibility of certain terms. After completion the participants were asked again whether, they had experienced difficulties in understanding the instruction, the three questions or a single word. In total, only one of the 46 contacted persons declined to participate in the pretest: a 56-year-old male engineer declined due to protection of privacy. The participation of a 46-year-old female cleaner was interrupted due to functional illiteracy.

The answered questionnaires were reviewed with regard to completeness and inconsistent findings. The instruction of the questionnaire asked the participants to provide information for *all* anatomical regions. Accordingly, if they had no pain or complaints in certain anatomical regions, the category "never" had to be checked when answering the question in the frequency scale “During the last work week how often did you experience ache, pain, discomfort?”. Omitted items were rated as missing data. If the subjects claimed to have experienced pain or constraints in the frequency scale (every category but “never”), they had to indicate corresponding information in the severity scale and work interference scale. Omitted items in the latter were rated as missing data as well.

Inconsistency was encountered when subjects claimed they did not feel pain or complaints in certain anatomical regions within the frequency scale (“never”) but indicated severity and work interference for the same anatomical regions.

Taken as a whole, none of the pretest’s subjects claimed that they had difficulties with regard to clarity and comprehensibility when filling out the questionnaire. In some cases, items were omitted when subjects felt no complaints and thus left out certain anatomical regions in the questionnaire. Omitted items were the most frequent cause of missing data. With 20 items and 44 participants there were 880 possible items. Missing data occured in only 2 % of all cases. Inconsistency was found in many more cases (15 %).

The most frequent error was found when subjects answered the questions within the work interference scale “If you experienced ache, pain, discomfort, does this interfere with your work ability?” with “not at all” although they had claimed earlier that they did not experience pain and discomfort. This, however, did not lead to a substantial contradiction. Knowing that the regional score, as suggested by Hedge, is calculated as the product (frequency x intensity x work interference) and “never” equates to the value “0”, this error had no consequence, because in this case the result would remain “0” [28]. For this reason, the expert committee decided against further modifications in this particular case.

Besides missing data and inconsistent findings, the study participants provided two further indications in the interviews. The first indication concerned the fact that pain in the elbow could not be directly assigned by the subjects. Furthermore, it was apparent that a subdivision of the hip/buttocks into right-side and left-side would have been useful. Both indications were been added to the final version of the D-CMDQ after they had been discussed within the expert committee and after consultation with the developer of the CMDQ. Finally, the illustration of the female body was slightly modified in order to visualize the neck area more precisely. Later the male version of the standing worker was adapted. By doing so, the cross-cultural adaption process was completed.

### Validity assessment

Numeric Rating Scales (NRS) have been reported to yield reliable, valid, and sensitive measurements of pain intensity [29]. In this study an 11-point NRS (0–10) was used as a criterion for current overall pain intensity to test the construct validity of the D-CMDQ. For that the correspondence between the two methods was proved. The NRS assesses pain intensity from 0 (no pain) to 10 (worst imaginable pain), and study participants were asked to check the correct number describing the worst pain perceived during the previous week for each body area. The participants were then asked to complete the D-CMDQ. The responses regarding the occurrence of pain were then compared between both questionnaires in the same subjects. It was also examined whether all participants who had reported pain in the NRS did so in the D-CMDQ frequency scale as well. Those participants who had declared “no pain” in the NRS were expected to check “never”in the D-CMDQ frequency scale. Further NRS scores were expected to correlate positively with D-CMDQ severity scores.

The agreement between responses given in NRS and the D-CMDQ frequency scale was analyzed by Kappa coefficients (κ). There are different recommendations for the interpretation of the Kappa statistic [30–32]. Following Landis & Koch [30] Kappa values between 0.61 and 0.80 were found to be substantial. Values between 0.00 and 0.20 were believed to be slight, values between 0.21 and 0.40 were believed to be fair, and values between 0.41 and 0.60 were regarded as moderate.

The Spearman rank correlation statistic was used to determine the correlation between NRS scores and D-CMDQ severity scale scores. Spearman’s correlation coefficients allow the analysis of the strength of association between variables of ordinal measurement levels in a single value between -1 (negative association) and +1 (positive association). The values can be interpreted as follows: very low association between 0.00 and 0.20; low association between 0.20 and 0.50; moderate association between 0.50 and 0.70; high association between 0.70 and 0.90; very high association between 0.90 and 1.00 [33]. These values are considered to be recommendations. The interpretation of a value always depends on the scientific question.

### Reliability assessment

Test-retest reliability for self-administrated tests is measured by presenting a questionnaire twice to a person separated by a given time interval in order to assess stability over time. In this study, a time interval of 7 days was used as a reasonable compromise between memory bias and clinical change [34, 35]. For sample size determination the general recommendation of 2 to 20 subjects per item of the instrument scale was used [36, 37].

First test-retest reliability was calculated by Spearman rank correlation coefficients using mean sum scores of each scale (frequency, severity scale, and work interference scale) [38]. In general, high correlations can be expected for time-stable attributes only. Additionally, Kappa coefficients were calculated to analyze the test-retest-reliability for the responses given on the frequency, the severity and the work interference scale for each body area separately. In musculoskeletal research, ratings for clinical diagnosis or classification often lie on an ordinal scale. For this data the kappa statistic is an appropriate measure of reliability, provides valuable information and is commonly used even in the clinical setting [39]. The Kappa value is influenced by the prevalence of the outcome [40] and depends on the number of categories. The CMDQ frequency scale includes 5 categories; the CMDQ severity and work interference scale include 3 categories, respectively. The prevalence effect is related to the probabilities of “yes” and “no” and can lead to low Kappa values in the calculation of Kappa statistics despite of high agreement. Thus in this study an heuristic approach was used to overcome this problem by calculating a maximum Kappa (κ_max_) for each measurement to compute the arithmetical ratio (κ/κ_max_) subsequently [41]. Kappa maximum is the highest obtainable agreement for a specific data set to relativize the Kappa correlation coefficient. Additionally, the proportion of observed agreement (P_O_) and the proportions of positive (p_pos_) and negative agreement (p_neg_) were calculated to obtain more information about response consistency (Table 3). p_pos_ is the number of positive responses agreed on for both measuring points divided by all positive responses for both tests, and likewise for p_neg_ [42].

Finally, the internal consistency of each scale was tested by calculating Cronbach’s alpha statistic. For empirical investigations an alpha-lower limit of 0.70 is considered as satisfactory [43].

### Ethical approval

All procedures performed in the study involving human participants where in accordance with the Helsinki declaration or comparable ethical standards. The design and protocol of the study was approved by the Ethics Committee of the University of Rostock. Participants were informed about the study purpose, methods, and confidentiality of data. Written informed consent was obtained from all participants included in this study.

### Statistics

All analyses were conducted using the IBM SPSS software, version 22.0 for Windows® (Statistical Package for the Social Sciences, Chicago, IL, USA). In all analyses, *P* values < 0.05 were considered as statistically significant.

## Results

### Sample

A convenience sample of 68 subjects of different professions participated in the validation process. All study participants were native Germans. The characteristics of the participants are shown in Table 1.

**Table 1:** Sociodemographic and work-related data of the subjects (*n* = 68)

Age (years)	Total	44.8 ± 12.6
	>29	9 (13,2)
	30–39	16 (23,5)
	40–49	19 (27,9)
	50–59	13 (19,1)
	≥60	11 (16,2)
Gender		
	Female	31 (45.6)
	Male	37 (54.4)
Education		
	Skilled worker	14 (20.6)
	Vocational school	26 (38.2)
	High school/technical school level	11 (16.2)
	College level/University	17 (25.0)
Professional situation		
	Mainly physical workload	25 (36.8)
	Mainly mental workload	43 (63.2)
Health condition		
	Musculoskeletal disorders	60 (88.2)
Prevalence of pain/complaints in the last week		
	Neck	26 (38.2)
	Shoulders	20 (29.4)
	Upper back	21 (30.9)
	Upper arm	7 (10.3)
	Lower back	37 (54.4)
	Forearm	6 (8.8)
	Wrists	11 (16.2)
	Hip/Buttocks	12 (17.6)
	Thighs	4 (5.9)
	Knees	15 (22.1)
	Lower leg	3 (4.4)
	Foot	12 (17.6)

Quantitative variables (age): mean ± standard deviation; categorical variables: frequency (percentage)

In the initial examination, 88.2 % of all 68 participants reported musculoskeletal pain and complaints in at least one body part. More than three-quarters of all participants specified pain and complaints in two body parts. Most frequent were complaints in the lower back (54.4 %), followed by complaints in the neck (38.2 %) and the upper back (30.9 %). Pain in the upper arm, elbow, thigh, and lower leg occurred rarely (<10 %).

For the test-retest reliability complete data sets of 48 participants were analyzed. None of the subjects reported a medical treatment or a change in medication between the two measurement points.

### Validity

Table 2 shows the results of the validity assessment. Kappa coefficients demonstrate the agreement between the responses given on the NRS and on the D-CMDQ frequency scale and ranged from 0.38 (right thigh) to 1.00 (right foot). In total 65 % of the items showed excellent, 30 % substantial, and only 4 % (one item) showed fair agreement The association between the responses given on the NRS and the D-CMDQ severity scale obtained by Spearman’s correlation coefficients ranged from 0.40 (right thigh) to 1.00 (right foot). In all 39 % of the items had very high and high agreement, respectively, 17 % showed moderate and 4 % (one item) showed only low agreement. All of these correlations were statistically significant (*p* < 0.01).

**Table 2:** Validity assessment results (*n* = 68)

Validity				
Body regions	Agreement between NRS & D-CMDQ	Strength of agreement^a^	Correlation between NRS & D-CMDQ	Strength of agreement^b^
	Frequency scale		Severity scale	
	Cohen’s Kappa		Spearman rank correl. coefficients	
Neck	0.97	excellent	0.97**	very high
Right shoulder	0.96	excellent	0.97**	very high
Left shoulder	0.96	excellent	0.97**	very high
Upper back	0.83	excellent	0.80**	high
Right upper arm	0.64	substantial	0.64**	moderate
Left upper arm	0.92	excellent	0.93**	very high
Lower back	0.94	excellent	0.89**	high
Right elbow	0.70	substantial	0.74**	high
Left elbow	0.65	substantial	0.70**	moderate
Right forearm	0.79	substantial	0.82**	high
Left forearm	0.66	substantial	0.70**	moderate
Right wrist	0.94	excellent	0.94**	very high
Left wrist	0.88	excellent	0.89**	high
Right hip/buttocks	0.84	excellent	0.80**	high
Left hip/buttocks	0.88	excellent	0.90**	high
Right thigh	0.38	fair	0.40**	low
Left thigh	0.65	substantial	0.67**	moderate
Right knee	0.91	excellent	0.96**	very high
Left knee	0.96	excellent	0.95**	very high
Right lower leg	0.79	substantial	0.80**	high
Left lower leg	0.85	excellent	0.86**	high
Right foot	1.00	excellent	1.00**	very high
Left foot	0.93	excellent	0.94**	very high

**p < 0.01; *NRS* Numeric Rating Scale, *D-CMDQ* German version of the Cornell Musculoskeletal Discomfort Questionnaire; ^a^ [30]; ^b^ [33]

### Reliability

Regarding the internal consistency, Cronbach’s alpha statistics for the frequency, the severity, and the work interference scales were 0.75, 0.77, and 0.82, respectively.

The test-retest reliability calculated by Spearman rank correlation coefficients using a sum score of each scale was 0.56, 0.72, and 0.72 for the frequency, the severity, and the work interference scale, respectively.

Table 3 shows the association between test and retest responses at the level of the body parts (agreement of responses). Despite a high proportion of observed agreement, low Kappa values which are affected by the relative probabilities of the “yes” and “no” categories were found. In the study presented, for the most body parts the number of subjects without pain was much higher than the number of subjects with pain. For all three scales, negative responses were more consistent on average (p_neg_ = 0.76–0.83) than positive responses (p_pos_ = 0.05–0.06). The calculation of the maximum Kappa was an approach to take into account the “prevalence effect” [44]. The table also shows that in some body parts with low prevalence (proportion of “yes” responses) the values of the Kappa coefficients were “0” with both methods. In all cases the variable at the second measurement point was a constant (all subjects reported “no pain”). For these body parts a relative Kappa (κ/κ_max_) could not be calculated. The proportion of excellent/substantial/moderate and poor strength of agreement was 29 %/24 %/41 % and 5 % for the frequency scale, 22 %/28 %/33 % and 11 % for the severity scale, and 22 %/39 %/28 % and 11 % for the work interference scale, respectively. For the severity scale additionally 5 % of the relative Kappa values were of fair strength.

**Table 3:** Test-retest reliability assessment results (*n* = 48)

	D-CMDQ					D-CMDQ					D-CMDQ				
	Frequency scale					Severity scale					Work interference scale				
Body region	p_pos_	p_neg_	P_O_	κ	κ/κmax	p_pos_	p_neg_	P_O_	κ	κ/κmax	p_pos_	p_neg_	P_Ο_	κ	κ/κmax
Neck	0.25	0.48	0.73	0.54	0.66	0.27	0.48	0.75	0.56	0.74	0.29	0.48	0.77	0.62	0.75
Right shoulder	0.17	0.63	0.79	0.49	0.49	0.17	0.62	0.79	0.46	0.51	0.19	0.63	0.81	0.58	0.61
Left shoulder	0.10	0.79	0.90	0.55	0.71	0.10	0.79	0.90	0.55	0.71	0.08	0.79	0.88	0.55	0.71
Upper back	0.17	0.60	0.77	0.44	0.46	0.10	0.63	0.73	0.40	0.49	0.13	0.63	0.75	0.43	0.45
Right upper arm	0.02	0.85	0.87	0.21	0.43	0.02	0.85	0.88	0.22	0.61	0.02	0.86	0.88	0.21	0.60
Left upper arm	0.00	0.94	0.94	*	*	0.00	0.94	0.94	*	*	0.00	0.94	0.94	*	*
Lower back	0.17	0.42	0.58	0.34	0.49	0.21	0.42	0.62	0.34	0.50	0.21	0.42	0.63	0.41	0.59
Right elbow	0.00	0.92	0.92	*	*	0.00	0.92	0.92	*	*	0.00	0.92	1.00	*	*
Left elbow	#	1.00	1.00	*	*	0.00	1.00	1.00	*	*	0.00	1.00	1.00	*	*
Right forearm	0.02	0.90	0.92	0.40	0.98	0.04	0.90	0.94	0.55	0.98	0.04	0.90	0.94	0.55	1.00
Left forearm	0.00	0.92	0.92	−0.21	−0.43	0.00	0.92	0.92	−0.16	−0.33	0.00	0.92	0.92	−0.16	−0.33
Right wrist	0.10	0.77	0.88	0.58	0.67	0.04	0.77	0.81	0.36	0.46	0.08	0.77	0.85	0.50	0.63
Left wrist	0.02	0.88	0.92	0.35	0.57	0.04	0.88	0.92	0.22	0.36	0.04	0.88	0.92	0.46	0.82
Right hip/buttocks	0.06	0.88	0.94	0.68	0.68	0.04	0.88	0.92	0.68	0.76	0.04	0.92	0.96	0.78	0.88
Left hip/buttocks	0.10	0.90	1.00	1.00	1.00	0.10	0.90	1.00	1.00	1.00	0.10	0.90	1.00	1.00	1.00
Right thigh	0.04	0.90	0.94	0.55	0.98	0.04	0.90	0.94	0.55	0.98	0.02	0.90	0.92	0.40	0.71
Left thigh	0.02	0.92	0.94	0.38	1.00	0.02	0.92	0.94	0.38	1.00	0.00	0.92	0.92	0.18	0.47
Right knee	0.08	0.79	0.88	0.55	0.60	0.08	0.79	0.88	0.55	0.71	0.06	0.79	0.85	0.47	0.51
Left knee	0.00	0.92	0.92	*	*	0.00	0.92	0.92	*	*	0.00	0.92	0.92	*	*
Right lower leg	0.00	0.98	0.98	*	*	0.00	0.96	0.96	*	*	0.00	0.96	0.96	*	*
Left lower leg	0.00	0.96	0.96	−0.01	*	0.00	0.96	0.96	−0.01	−0.01	0.00	0.96	0.96	−0.02	−0.02
Right foot	0.06	0.90	0.96	0.73	0.85	0.02	0.90	0.92	0.46	0.53			0.96	0.73	0.79
Left foot	0.02	0.90	0.92	0.40	0.57	0.02	0.90	0.92	0.39	0.46			0.94	0.55	0.65
Mean	0.06	0.83	0.89	0.44	0.63	0.05	0.83	0.89	0.42	0.58	0.06	0.76	0.90	0.46	0,57

*D-CMDQ* German version of the Cornell Musculoskeletal Discomfort Questionnaire, *κ* Cohen’s Kappa, *κ/κmax* proportion of maximum kappa achieved, *p*_*pos*_ proportion of positive agreement, *p*_*neg*_ proportion of negative agreement, *P*_*O*_ proportion of observed agreement; * Variable at the second measurement point was a constant (all subjects reported “no pain”). For these body regions a relative Kappa (κ/κmax) could not be calculated. # p_pos_ could not be calculated as nil positive responses on either testing occasion

## Discussion

The current study presents a cultural adaptation of the English version of the CMDQ into German, following internationally respected methodological procedures, and finally the validation of the D-CMDQ.

The results of the pre-test indicate that the translated and adopted D-CMDQ meets the essential requirements for clarity and comprehensibility for persons with different educational and occupational background. This is a basic precondition for a universal application of a self-administrated questionnaire at the workplace. The percentage of missing data and inconsistent responses was found to be acceptable.

Regarding the psychometric properties, the D-CMDQ demonstrated good validity: with the exception of the right thigh, the Kappa values of all body areas achieved the substantial range of agreement and 65 % of all values were found to be excellent [30]. In the study presented thighs belonged to those body parts for which the prevalence of symptoms was very low (about 5 %). Therefore, differences between the responses in the frequency scale of the D-CMDQ and the Numeric Rating Scale had a stronger effect on the Kappa value than for those body parts with higher prevalence rates. In terms of the validity of the severity scale, Spearman correlation coefficients of all body areas showed a marked and significant association to the NRS (except the right thigh again) and in 74 % of the cases a high to perfect correlation was found. These results are comparable with the published data of the Turkish version of the CMDQ [23].

The internal consistency of the D-CMDQ in this study was satisfactory with Cronbach’s alpha values of 0.75 for the frequency scale, 0.77 and 0.82 for the severity and work interference scale. Nevertheless, the published values of Cronbach’s alpha in the validation study of the Turkish version were higher (α = 0.88–0.89). In a recently conducted reapplication of the D-CMDQ in a sample of forestry workers (*n* = 88) alpha values of 0.88, 0.81, and 0.88 were found for the frequency, the severity and the work interference scale, respectively. The differences in the values of Cronbach’s alpha suggest that the internal consistency was influenced by the characteristics of the investigated sample. The participants of the Turkish validation study were workers of a metal manufacturing company and their physical workload is comparable to that of forestry workers rather than to that of an accidental sample, which is dominated by professions with mainly mental workload as in this study.

Test-retest reliability of the D-CMDQ calculated by the proportion of observed agreement (P_O_) of each scale indicated a markedly association. Nevertheless, the item level high agreement of responses did not correspond with high Kappa values.

Since the introduction of the Kappa statistic, some difficulties (paradoxes) associated with its interpretation have been described [40, 44, 45]. Originally, the Kappa statistic had been proposed for two observers scoring individuals as either positive or negative. Later the method was extended for multiple observers and more than two categories. In the case of more than two categorical properties (e.g., five within the CMDQ frequency scale), the opportunities for disagreement increase resulting in a lower Kappa value [46]. Looking at the proportion of non-identical responses of each scale in this study it becomes apparent that in 65 to 70 % the disagreement between two measures is 10 % or lower. Certainly for the three body regions with the highest prevalence of pain or complaints (lower back, upper back, neck) the proportion of disagreement is much higher (0.23–0.42). This is corresponding with lower Kappa values (fair to moderate strength of agreement) und could reduce the utility of the instrument.

For example, in comparison with CMDQ the Nordic questionnaire for the analysis of musculoskeletal symptoms [16] has only two categories for the assessment of symptoms in different body areas (yes/no). Therefore, the calculation of the test-retest reliability results in higher Kappa values. To overcome this problem a weighted Kappa statistic (κ_w_) has been proposed [47]. For our data the calculation of a weighted Kappa did not lead to a relevant improvement in the Kappa values but the prevalence effect [44] achieved relevance.

The difference between the probabilities of “yes” and “no” referred to as the prevalence index [40] affects the Kappa value. The larger the value of the prevalence index, the smaller is Kappa (ibid.). For the data of the present study a calculation of the maximum Kappa was carried out to relativize the bias of prevalence according to the recommendations of Xier [41]. This method resulted in Kappa values in parts comparable with the results of the validation study of the Turkish version [23]. In that study Kappa coefficients for the test-retest reliability ranged between 0.56–0.95, 0.56–0.97 and 0.59–0.94 for the frequency, the severity, and the work interference scale, respectively. The participants of the Turkish validation study were workers of a manufacturing company, 81.3 % of them were male. It might be that the subjects in this sample had a larger percentage of musculoskeletal symptoms and complaints, resulting in a smaller prevalence index and higher Kappa values. Indeed, the published data were not specified regarding this assumption.

In summary it can be stated that for categorical data the interpretation of a single coefficient of agreement is difficult. For comparisons between agreement studies sometimes a more pragmatic approach is essential and in the case of Kappa statistics observed agreement should be discussed as well as bias and prevalence.

The sample size and the prevalence of pain and complaints seem to be a major limitation of this study. Prevalence rates close to 50 % were recommended [48] to overcome the paradoxical effects of high and low prevalence of the kappa coefficient. But even in physically demanding occupational groups workers do not report such high prevalence rates of pain and complaints in all body regions [49]. Therefore, the limitations of Kappa statistics will persist in future. An augmented ranking approach to evaluate systematic and individual disagreement was developed by Svensson [50] to provide valuable interpretable information in paired ordinal assessments. This and other methods should apply more often in future studies to gain experience with new approaches for analysis and interpretation of data in the field of reliability assessment.

Other limitations of the study should be discussed: To avoid difficulties in the understanding of the instruction, the items, and the responses due to a lack of linguistic competence the study included only native Germans. Nevertheless, in future application we see no restriction to non-native German-speaking people in view of the linguistic clarity and simplicity. The illustration of the body areas and the tabulation essentially support the completion of the questionnaire.

Referring to the original English CMDQ, the presentation of the D-CMDQ also includes a female and a male version. This implies that a “third gender” is not taken into account and this affects the usefulness of the questionnaire. For further application a neutral illustration comparable with that used for the Nordic Musculoskeletal Questionnaire should be discussed.

Diverging from the guidelines for the process of cross-cultural adaptation of self-report measures [25] a German physician, who has lived as long in Germany as in the United States was consulted for the backwards translation . In consideration of the fact that phrases used in the questionnaire are more universal than regard medical content we feel confident that this variation of the guidelines is acceptable.

## Conclusions

Our results indicate that the adapted CMDQ is an appropriate method for the assessment of musculoskeletal disorders in the German-speaking work-force. The psychometric properties of the D-CMDQ meet the requirements of validity and reliability. The questionnaire is characterized by clarity and comprehensibility, and the possibility of universal application in different occupational groups.
